# Multiple Distinct Forms of CD8+ T Cell Cross-Reactivity and Specificities Revealed after 2009 H1N1 Influenza A Virus Infection in Mice

**DOI:** 10.1371/journal.pone.0046166

**Published:** 2012-09-27

**Authors:** Hailong Guo, David J. Topham

**Affiliations:** 1 Center for Infectious Diseases and Vaccine Immunology, Rochester General Hospital Research Institute, Rochester, New York, United States of America; 2 New York Influenza Center of Excellence, Department of Microbiology and Immunology, and the David H. Smith Center for Vaccine Biology and Immunology, Aab Institute of Biomedical Sciences, University of Rochester, Rochester, New York, United States of America; UC Irvine Medical Center, United States of America

## Abstract

Influenza primed mice are protected against lethal infection with H1N1 A/CA/04/E3/09 virus, and T depletion and serum transfer studies suggest a T-dependent mechanism. We therefore set out to investigate the quality of the cross-reactive T cell response to CA/E3/09 in mice primed with H3N2 influenza A/Hong Kong/X31 virus. Sequences of the immunodominant nucleoprotein (NP) NP366–374 and acid polymerase (PA) PA224–233 CD8 epitopes from X31 each differ from the CA/E3/09 virus by one amino acid: an M371V substitution at position 6 of the NP peptide, and an S224P substitution at position 1 of the PA peptide, raising questions about the role of these epitopes in protection. PA224–233 peptides from either virus could elicit IFN-γ spot forming cells from mice infected with X31, indicating cross-reactivity of these two peptides. However, no T cell responses to either PA224–233 peptide were detectable after primary CA/E3/09 infection, suggesting it is cryptic in this virus. In contrast, primary responses to the NP366 peptides were detectable after infection with either virus, but did not cross-react in vitro. Similarly, H2-D^b^ tetramers of each NP epitope stained CD8+ T cells from each respective virus infection, but did not obviously cross-react. Early after lethal CA/E3/09 challenge, X31 primed mice had enhanced IFN-γ responses toward both NP366 peptides, as well as recall responses to a set of subdominant NP and PA peptides not detectable after primary X31 infection alone. Furthermore, dual-tetramer staining revealed an expanded population of CD8 T cells reactive to both NP366 variant peptides also not seen after the priming infection alone. These observations demonstrate unusual CD8+ T cell cross-reactivity and specificity are elicited after primary and secondary CA/E3/09 influenza virus infections.

## Introduction

Through antigenic drift and shift, influenza virus actively changes the hemagglutinin (HA) and neuraminidase (NA) membrane proteins ostensibly to evade pre-existing humoral immunity. However, homo- and hetero-subtypic protection against variant influenza viruses could be achieved by cross-reactive CD8 T cell responses against influenza internal proteins. Thus generation of protective cross-reactive T cell immunity via immunization strategies remains as one possible approach for the development of broad cross-reactive or universal influenza vaccine [1].

In C57BL/6 mice, two major immunodominant H2-D^b^ restricted CD8 epitopes, NP366–374 and PA224–233 have been identified on nucleoprotein (NP) and acid polymerase (PA) proteins [2], [3] and extensively characterized using the well-known PR8 (H1N1) and X31 (H3N2) priming and challenge model [4], [5], [6], [7]. Although they differ in the HA and NA proteins, PR8 and X31 virus share the same 6 internal proteins, and priming with either virus protects against infection with the other virus. During primary infection, the responses of CD8 T cells specific for NP366 and PA224 are both prominent. However, during secondary challenge, the NP366 specific population accounts for the largest portion of the total virus-specific response, ranging at least 5 fold greater in magnitude than the next largest PA224 specific CD8 T cell population [3], [8]. Thus, NP and PA peptide specific CD8 cells play a crucial role in controlling influenza virus during primary and secondary infection. Although the internal proteins are relatively stable, mutation of these proteins, such as NP can occur via antigenic drift [9], [10]. Experimentally designed mutations of specific amino acid residues on NP366 and PA224 have been shown to impair or disrupt TCR recognition [3], [11], [12]. Further, under extreme CD8+ T cell immune pressure, virus variants that have mutated the immunodominant epitopes can readily emerge [9], [10], [13], indicating the potential ability of influenza virus to escape CD8 immunity.

**Table 1:** Predicted H2-Db restricted NP CD8 epitopes for CA/E3/09.

X31 NP			CA/E3/09 NP		
Position	Peptide	Affinity(nM)	Position	Peptide	Affinity(nM)
55	RLIQNSLTI	34	55	RLIQNSITI	17
366	ASNENMETM	58	366	ASNENVETM	89
39	**FYIQMCTEL**	179	39	**FYIQMCTEL**	179
296	**YSLVGIDPF**	1266	305	KLLQNSQVV	233
164	**CSLMQGSTL**	1476	296	**YSLVGIDPF**	1266
181	AAVKGVGTM	1517	164	**CSLMQGSTL**	1476
188	TMVMELVRM	1932	373	TMDSNTLEL	1911
41	**IQMCTELKL**	3138	181	AAVKGVGTI	2014
458	FQGRGVFEL	3899	130	TAGLTHIMI	2818
412	FSVQRNLPF	4271	41	**IQMCTELKL**	3138

X31 and CA/E3/09 NP amino acid sequences [18] were introduced into NetMHC 3.0 for H2-Db 9 mer CD8 epitope analysis. Listed are the top 10 peptides with higher MHC binding potential. Bolded peptides indicated identical sequences shared between CA/E3/09 and X31 viruses.

**Table 2:** Predicted H2-Db restricted PA CD8 epitopes for CA/E3/09.

X31 PA			CA/E3/09 PA		
Position	Peptide	Affinity (nM)	Position	Peptide	Affinity (nM)
224	SSLENFRAYV	131	69	**NALLKHRFEI**	307
69	**NALLKHRFEI**	307	450	**VSHCRATEYI**	567
450	**VSHCRATEYI**	567	439	ASMRRNYFTA	1520
643	**KSVFNSLYAS**	1619	643	**KSVFNSLYAS**	1619
564	**YVRTNGTSKI**	1754	564	**YVRTNGTSKI**	1754
509	**SHLRNDTDVV**	1911	509	**SHLRNDTDVV**	1911
467	**TALLNASCAA**	2011	467	**TALLNASCAA**	2011
508	**RSHLRNDTDV**	2865	224	PSLENFRAYV	2823
439	ASMRRNYFTS	3210	508	**RSHLRNDTDV**	2865
432	**VAPIEHIASM**	4200	432	**VAPIEHIASM**	4200

X31 and CA/E3/09 PA amino acid sequences [18] were introduced into NetMHC 3.0 for H2-Db 10 mer CD8 epitope analysis. Listed are the top 10 peptides with higher MHC binding potential. Bolded peptides indicated identical sequences shared between CA/E3/09 and X31 viruses.

2009 pandemic H1N1 influenza virus is a novel triple reassortant pathogenic influenza virus containing genes of avian, swine and human origins [14]. Cross-reactive CD4 and CD8 cells against the recent H1N1 virus could be detected in human peripheral blood samples collected before the pandemic [15], [16], [17], suggesting that this form of immunity may offer some protection from severe disease. Our recent study has shown that a laboratory variant of this novel H1N1 virus (CA/E3/09) is pathogenic in C57BL/6 mice [18], providing an alternative model for studying immune protection against this new influenza virus. Using this model, we have shown that homologous or heterologous protection against lethal infection of 2009 pandemic H1N1 virus, CA/E3/09, could be achieved by priming with homologous and heterologous isolates including PR8 and X31 viruses and this protection is dependent on CD4 and CD8 T cells [18], and is supported by other related recent studies [19], [20], [21]. Taken together, these data indicated the existence of cross-reactive T cell epitopes shared between 2009 pandemic H1N1 and distally related influenza viruses.

In the present study, we used bioinformatic and experimental approaches to predict and characterize potential CD8 epitopes on the NP and PA proteins of CA/E3/09 virus in comparison with X31 virus. We have found that the predicted NP366 and PA224 CD8 epitopes on CA/E3/09 virus, CANP366 and CAPA224, had one substitution each that resulted in the generation of new NP366 specific CD8 population that had minimal cross-reactivity with NP366 epitope derived from X31 virus and the loss of immunodominance of PA224 peptide. Moreover, infection with CA/E3/09 virus generated new subdominant and minor CD8 epitopes that were not presented during X31 infection. However, priming with X31 virus and re-challenge with CA/E3/09 resulted in generation of CD8 T cells that could cross-react with NP366 peptides from both CA/E3/09 and X31 viruses.

**Figure 1:**
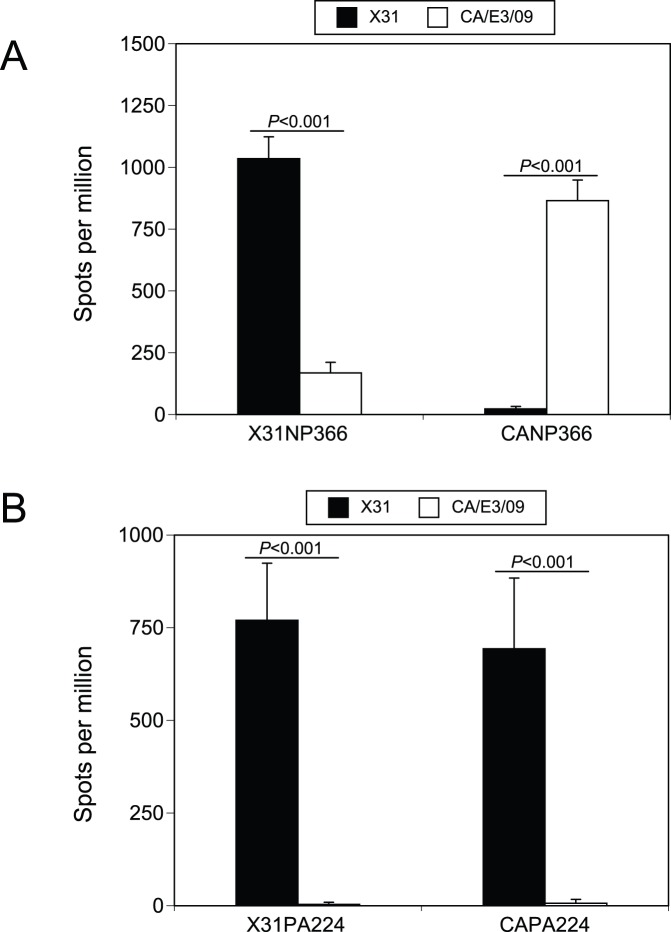
NP366 and PA224 peptide specific responses after CA/E3/09 infection. IFN-γ ELISPOT assay as described in the methods was used to detect NP366, X31NP366 and CANP366, (A) and PA224, X31PA224 and CAPA224, (B) specific CD8 responses at day 10 after naïve mice were non-lethally infected with X31 virus (3×10^5^ EID_50_, filled column) or CA/E3/09 virus (3 PFU, open column). Data presented were average values ± SD from 5 mice of each group and representative of at least four independent experiments.

## Results

### Predicted CD8 Epitopes for CA/E3/09 NP and PA Proteins

To search for potential CD8 epitopes in the NP and PA proteins of 2009 pandemic H1N1 influenza virus, we used the NetMHC 3.0 CD8 epitope prediction algorithm to analyze H2-D^b^ restricted CD8 epitopes on the NP and PA proteins of CA/E3/09 virus. The NP366 and PA224 CD8 epitopes previously identified during X31 or PR8 infection in B6 mice were 9 and 10 mers, respectively, so we focused on screening 9 mer NP peptides and 10 mer PA peptides using the deduced NP and PA amino acid sequences of CA/E3/09 virus [18]. The NetMHC 3.0 algorithm generated a total of 490 predicted H2-D^b^ restricted CD8 peptides for both CA/E3/09 and X31 NP proteins and 707 potential H2-D^b^ restricted CD8 peptides for both CA/E3/09 and X31 PA proteins (data not shown). In Tables 1 and 2, we list 10 peptides from each of these proteins showing highest predicted potential to bind with H2-D^b^. Among the NP peptides, 4 were fully conserved between X31 and CA/E3/09 virus (Table 1), while in the PA peptides, X31 and CA/E3/09 virus shared 8 (Table 2).

**Figure 2:**
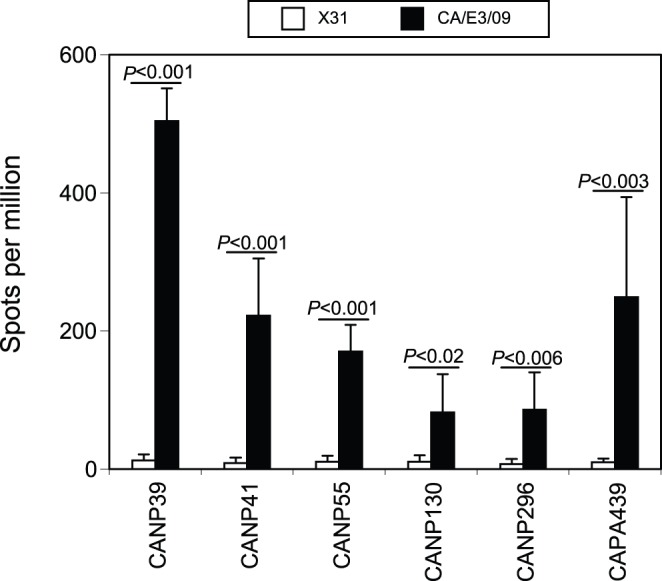
IFN-γ response to five new CD8 peptides after CA/E3/09 infection. A panel of predicted CD8 peptides excluding the NP366 and PA224 as indicated in the Table 1 and 2 were assessed for their abilities to induce IFN-γ response at day 10 after naïve mice were non-lethally infected with X31 virus (3×10^5^ EID_50_, filled column) or CA/E3/09 virus (3 PFU, open column) using the ELISPOT methods. Among these peptides, only five (CANP39, CANP41, CANP55, CANP130, CANP296 and CAPA439) were able to induce spots formation after CA/E3/09 infection. Data presented were average values ± SD from 5 mice of each group and representative of at least four independent experiments.

**Figure 3:**
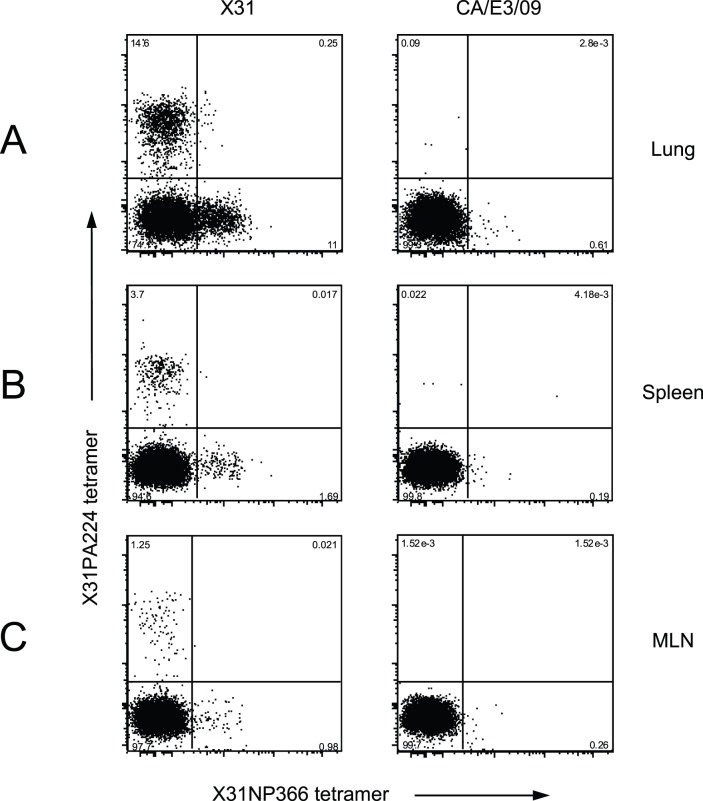
Detection of peptide specific CD8 cells after X31 and CA/E3/09 infection. Mice were non-lethally infected with X31 (3×10^5^ EID_50_) or CA/E3/09 virus (3 PFU). At day 10 of infections, CD8 antibody and H2-Db restricted X31NP366 and PA224 tetramers were used to detect peptide specific CD8 cells in live tissue cells from lung (A), spleen (B) and MLN (C) pooled from five mice. Data are representative of the results for at least three independent experiments.

**Figure 4:**
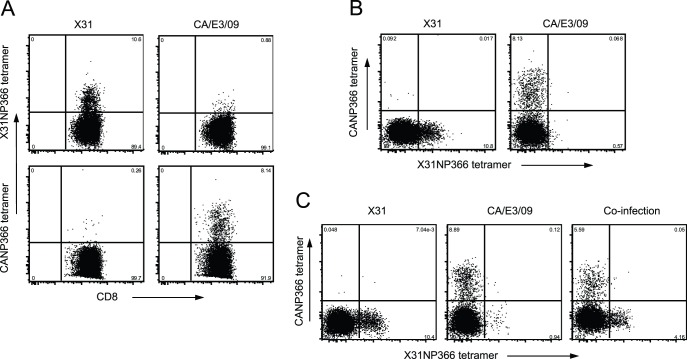
CANP366 specific CD8 cells revealed by new CD8 tetramer. Mice were non-lethally infected with X31 (3×10^5^ EID_50_) or CA/E3/09 virus (3 PFU) or a mixture of X31 and CA/E3/09 virus containing 3×10^5^ EID_50_ X31 and 3 PFU of CA/E3/09. At day 10 of infections, CD8 antibody and the newly generated H2-Db restricted tetramer specific for CANP366 were used to detect CA/E3/09 virus specific CD8 cells. Lung cell samples from X31 and CA/E3/09 infected mice were individually stained for X31NP366 and CANP366 tetramers (A) or co-stained with both NP366 tetramers (B). In addition, lung cell samples from X31 and CA/E3/09 co-infected mice were co-stained with X31NP366 and CANP366 tetramers (C). Data are representative of the results for two independent experiments.

**Figure 5:**
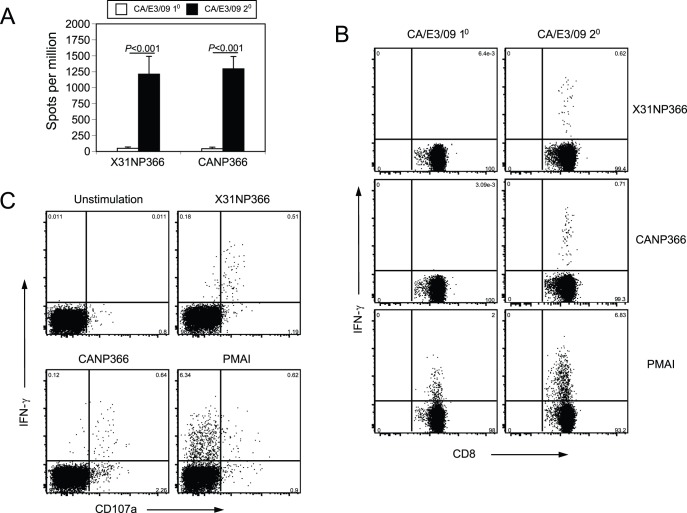
IFN-γ responses to NP366 peptides after rechallenge. Mice were primed with X31 virus (3×10^5^ EID_50_) and rested for 42 days before challenged with a lethal dose of CA/E3/09 virus (3000 PFU). At day 6 after the rechallenge, the IFN-γ responses for X31NP366 and CANP366 were measured by ELISPOT assay (A). In addition, after in-vitro NP366 peptide or PMAI stimulation, intracellular staining was used to measure IFN-γ production with or without X31 priming (B). At day 6 after the rechallenge, NP366 peptide or PMAI stimulated cell samples were also stained for CD107a before intracellular IFN-γ staining (C). Data are representative of two independent experiments.

**Figure 6:**
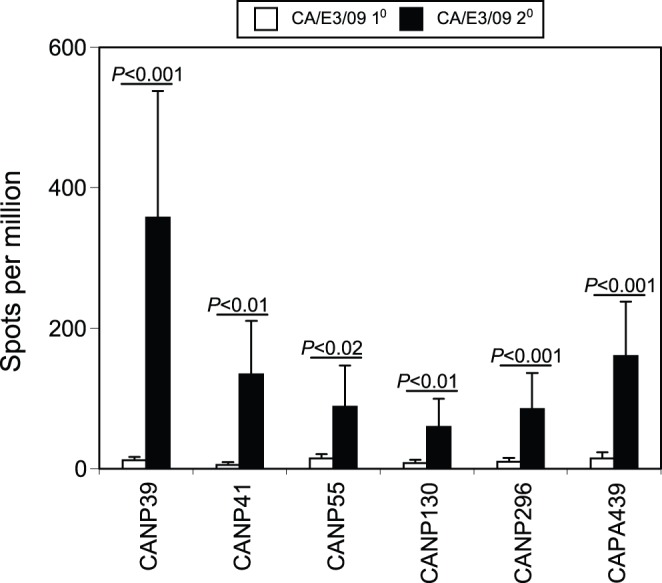
Immune responses to the newly identified CD8 peptides after rechallenge. Mice were primed with X31 virus (3×10^5^ EID_50_) and rested for 42 days before challenged with a lethal dose of CA/E3/09 virus (3000 PFU). At day 6 after the rechallenge, the IFN-γ responses for identified CA/E3/09 CD8 peptides including CANP399, CANP41, CANP55, CANP130, CANP296 and CAPA439 were measured by ELISPOT assay (A) Data are representative of three independent experiments.

From the peptide prediction lists, we found both CA/E3/09 and X31 derived NP366 peptides (CANP366 and X31NP366) ranked second based on predicted H2-D^b^ binding affinity, although CANP366 had a slightly lower predicted D^b^ binding affinity compared to X31NP366 (Table 1). Thus, the data suggested the potential dominance of the CANP366 epitope during infection with CA/E3/09 virus, similar to X31NP366 during X31 infection. The NP55, which had highest predicted affinity, has been identified previously but has been negative in assays [22], [23]. For the PA epitopes, we observed X31 PA224 peptide (X31PA224) had highest potential affinity, yet for CA/E3/09 derived PA224 peptide (CAPA224), the predicted affinity is substantially reduced (Table 2). Each of the CANP366 and CAPA224 epitopes from CA/E3/09 virus have one substitution in peptide position 6 and 1, M371V and S224P respectively, which may affect both potential H2-Db binding affinity and T cell recognition.

**Figure 7:**
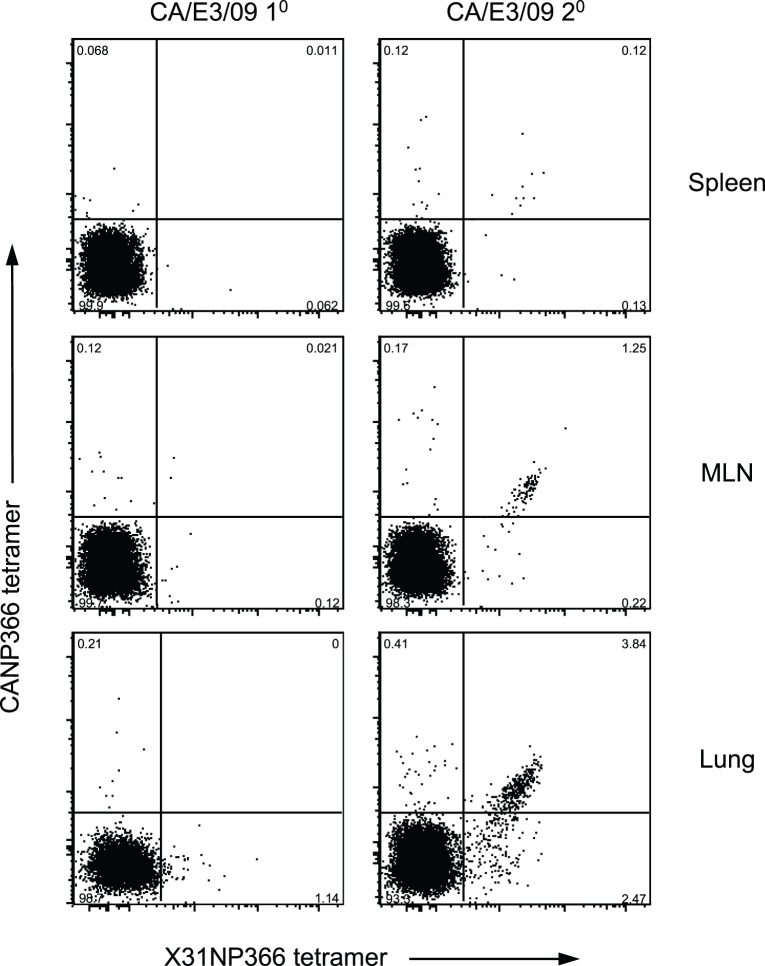
Detection of cross-reactive NP366 CD8 population after secondary challenge. Mice were primed with X31 virus (3×10^5^ EID_50_) and rested for 42 days and then were challenged with a lethal dose of CA/E3/09 virus (3000 PFU). At day 6 after re-infection, co-staining with X31NP366 and CANP366 tetramers were performed on cell samples obtained from spleen (A), MLN (B) and lung (C). Data are representative of three independent experiments.

### Altered NP366 and PA224 Repertoires during Primary H1N1 CA/E3/09 Influenza Infection

In order to examine whether the CA/E3/09 infection generates responses to the predicted CD8 epitopes, we synthesized the 28 peptides listed in Table 1 and 2, which included a total of 12 peptides identical between X31 and CA/E3/09 virus, and used IFN-γ ELISPOT assay to detect T cell responses. As expected, X31NP366 and X31PA224 could induce robust IFN-γ in vitro responses at day 10 after either X31 (Fig. 1A and 1B) or PR8 infection (data not shown). However, single M371V replacement in the CANP366 significantly disrupted the peptide’s ability to induce IFN-γ production by CD8 cells from X31 infected mice (Fig. 1A). In contrast, after stimulation with CANP366, cells from CA/E3/09 virus infected mice displayed strong IFN-γ responses equivalent to the anti-X31NP366 IFN-γ production in X31 infected mice (Fig. 1A). Unlike the CANP366, CAPA224 could stimulate IFN-γ response by cells from X31 infection to a similar extent as X31PA224 peptide (Fig. 1B), indicating X31-primed T cells can cross-react with the two variant peptides. However, there was no IFN-γ production by CD8 cells from CA/E3/09 virus infected mice in response to either PA224 peptide (Fig. 1B), suggesting that the CAPA224 epitope is not immunogenic in this infection. Together the data show that in spite of similarities, each virus infection alone elicits NP366-specific CD8+ T cells that are not cross-reactive, and that PA224-specific CD8+ T cells that cross-react are elicited only after X31 infection.

In contrast to the absence of the PA224 CD8 cells after CA/E3/09 infection, we could detect IFN-γ responses to a number of other peptides, conserved and non-conserved between X31 and CA/E3/09 viruses (Fig. 2). The highest frequency of cells responded to the CANP39 peptide (Fig. 2), although the frequency was still lower than that stimulated by CANP366 peptide (Fig. 1 and Fig. 2). CA/E3/09 and X31 viruses have identical amino acid sequence for the NP39 peptide (Table 1), yet the IFN-γ response against NP39 was not detected after X31 infection (Fig. 2). In addition to NP39, several other peptides from CA/E3/09 NP and PA proteins (CANP41, CANP55, CANP130, CANP296 and CAPA439) could also consistently stimulate relatively weak IFN-γ responses from animals infected with CA/E3/09, but not X31 virus (Fig. 2). No detectable responses were observed for other peptides listed in the Table 1 and 2 after either X31 or CA/E3/09 infection. Taken together, the results indicated that CA/E3/09 infection in B6 mice generates a novel CD8 epitope profile compared to X31 infection in spite of several fully conserved epitopes predicted.

### Mutations in PA224 and NP366 Affect Peptide-MHC Recognition

The impaired ability of CANP366 to induce IFN-γ response after infection with X31 virus and the absence of anti-CAPA224 IFN-γ response after CA/E3/09 infection indicated the amino acid changes on the predicted CA/E3/09 NP366 and PA224 CD8 epitopes affects the generation of peptide specific CD8 T cells. To investigate further, we used H2-D^b^ restricted X31NP366 and X31PA224 peptide specific CD8 tetramers for detecting peptide specific CD8 T cells. As seen in Fig. 3A, 10 days after X31 infection, both X31NP366 and X31PA224 peptide specific CD8 T cells in the lung tissues reached high frequencies as expected. However, with cells from CA/E3/09 infected mice, staining with X31NP366 and X31PA224 tetramers was low or at background levels (Fig. 3A, Fig. 3B, Fig. 3C). These results and the IFN-γ ELISPOT data reinforce the conclusion that while the NP366 epitope is immunogenic in both viruses, NP366 specific primary CD8 cells are minimally cross-reactive. The data also show that while the CAPA224 peptide can stimulate X31PA224 specific T cells in an ELISPOT assay, the affinity of these T cells for the CAPA224/D^b^ complex is likely too low for tetramer binding.

To detect CANP366 specific CD8 T cells, we prepared CANP366 Db tetramers. As seen in Fig. 4A, at 10 days after CA/E3/09 infection, the CANP366 D^b^ tetramer detected specific CD8 cells that comprised about 8.0% of the total lung CD8 T cells. In addition, CANP366 tetramer positive cells were also detected in spleen and MLN from CA/E3/09 infected mice (data not shown). As expected, there was little to no CANP366 tetramer staining of cell samples from X31 infected mice, while the X31NP366 tetramer specific cells were easily detected (Fig. 4A). Co-staining of lung samples from either CA/E3/09 or X31 infected mice using X31NP366 and CANP366 tetramers revealed mutually exclusive staining (Fig. 4B). Further, in a separate experiment, co-infection with X31 and CA/E3/09 virus resulted in a similar pattern of distinct X31NP366 and CANP366 tetramer specific CD8 T cells (Fig. 4C), though X31NP366 tetramer staining of cells from CA/E3/09 infected mice showed a slightly higher frequency (0.94 vs 0.57%). Thus, the tetramer staining data presented here further supported the observation that the single M371V substitution in the CA/E3/09 virus results in minimal cross-reactivity between the related epitopes, though it remains immunogenic.

### Early CD8 Cell Response after Secondary Challenge

We have recently demonstrated that influenza X31 priming could provide protection against lethal challenge of CA/E3/09 virus [18]. The protective effects became evident at day 6 after re-challenge when X31 primed mice start to regain body weight, while non-primed mice continue to lose weight [18]. Depletion studies indicated that both CD4 and CD8 T cells contribute to this heterosubtypic protection. T cell protection against heterosubtypic influenza has typically been explained by the recognition of conserved and therefore cross-reactive epitopes [24], [25]. However, so far, our data have suggested that the NP and PA peptides derived from the CA/E3/09 or X31/PR8 viruses, and predicted to bind MHC, have no profound cross-reactivity after primary infection. It is possible that cross-reactive epitopes exist in other viral proteins. One other possibility is that prior X31 virus priming promotes the production of a minority population of cross-reactive NP and/or PA CD8 T cells that can undergo robust expansion after CA/E3/09 rechallenge. If this is the case, we would be able to detect these cross-reactive CD8 T cells and their functional activations earlier after re-infection. Substantial peptide-specific CD8+ T cells responses can often be detected within 6 days of a secondary influenza infection, while taking up to 8 days to become robustly detectable after primary infection. Thus, we examined the responses to the CD8 epitopes on CA/E3/09 NP and PA proteins during secondary infection of X31 primed mice. As seen in Fig. 5A, at day 6 after secondary challenge, mice primed with X31 virus showed robust frequencies of CD8+ T cells reactive with CANP366 peptide compared to mice that were not primed (Fig. 5A). Interestingly, unlike in the primary infection, we also observed IFN-γ production in the presence of X31NP366 peptides at a level similar to CANP366 stimulation (Fig. 5A). We further performed intracellular staining after 4 hr peptide stimulation in vitro at day 6 and found that CD8 T cells from X31 primed mice produced similar amount of IFN-γ when stimulated with either CANP366 or X31NP366 peptide (Fig. 5B), but not after primary infection, confirming the observation using the ELISPOT methods. Many of these cross-reactive cells also expressed CD107a, a marker of cytolytic granules [26], [27] (Fig. 5C). This suggests that after rechallenge there is an expansion of cross-reactive NP366 specific CD8+ T cells that are functionally competent.

We also examined the responses to the additional NP and PA CD8 epitopes identified in the CA/E3/09 virus (Tables 1 and 2, Fig. 2). After secondary challenge, there were high frequencies of IFN-γ producing CD8 T cell responses against CANP39, CANP41, CANP55, CANP130, CANP296 and CAPA439 peptides detected at day 6 in X31 primed mice than naïve mice (Fig. 6). This was surprising given that responses to these peptides in primary X31 infection were at or below the limits of detection. Yet the accelerated kinetics of the responses to these peptides after secondary infection with the CA/E3/09 virus suggests that T cells reactive with these epitopes may have been primed in the first infection.

### Emergence of Cross-reactive NP366 CD8 Cells after Secondary Challenge

One interesting observation for the secondary CD8 peptide responses is the equal IFN-γ responses to X31NP366 and CANP366 (Fig. 5). The data indicated that prior priming with X31 virus increases the pool of individual X31NP366 and CANP366 CD8 T cell populations available during secondary lethal challenge. Alternatively, it is possible that priming with X31 and challenge with CA/E3/09 virus generates CD8 T cells that recognize both X31NP366 and CANP366 epitopes. To distinguish these possibilities, we used H2-D^b^ restricted CD8 tetramers specific for X31NP366 and CANP366 peptides to co-stain the tissue samples collected at day 6 from rechallenged mice. Our tetramer co-staining data revealed substantial frequencies of double positive CD8+ T cell populations in spleen, MLN and lung tissue from secondarily infected mice, but not those experiencing a primary infection (Fig. 7). This observation indicates that prior priming facilitated the expansion of CD8 cells that truly cross-react with both X31NP366 and CANP366 peptides.

## Discussion

We have observed at least four types of CD8+ T cell recognition in the context of these influenza infections. Type 1 was CD8+ T cell responses to epitopes that are unique to each virus infection. Type 2 was CD8+ T cells that recognize both WT and mutated peptides. Type 3 was T cells that recognize related, but mutated peptides from each virus, but in a mutually exclusive manner. Lastly, in influenza rechallenged mice, we observed CD8+ T cells with dual specificity not evident in the primary infections. These observations reveal tremendous plasticity in the ability to develop cross-reactive CD8+ T cells after influenza infection.

In PR8 and X31 infection B6 mouse model [4], [5], [6], [7], H2-D^b^ restricted CD8 epitopes NP366 and PA224 are dominant during primary infection, while NP366 is predominant during secondary infection [3], [8]. Although these studies provided convincing data for the dominance of certain influenza CD8 epitopes, characterization and validation of CD8 epitope hierarchy in other influenza virus infections is less well studied, possibly due to the fact that most natural influenza isolates could not easily infect animals. Recently, we have derived a 2009 H1N1 pandemic influenza viral stock, CA/E3/09 that can easily infect mice and cause severe lung disease without further adaption in mice [18]. Taking advantage of this new viral isolate, in this study, CD8 epitope profiles for NP and PA proteins of CA/E3/09 virus were analyzed with the aid of bioinformatic prediction and experimental approaches. However, it was not our intent in this study to conduct a full survey for all possible epitopes.

Our data indicate that, although it has valine substituted at position 6 for the methionine, the predicted CANP366 epitope is a dominant CD8 epitope processed and presented during CA/E3/09 infection in B6 mice. It has been shown that M6 residue on NP366 epitope of PR8 or X31 virus is critical for TCR recognition, as single M6A mutations results in complete loss of peptide-induced cytokine production and cytotoxicity [11], [28]. Interestingly, the virus carrying M371A mutation maintained NP366 immunodominance during infection [11]. Similar to the laboratory M371A mutation, the M371V substitution on CA/E3/09 NP protein also dramatically changed the antigen specificity of the native epitope, yet the CANP366 epitope is still dominant among other epitopes identified. Thus, our data and the previous reports suggested that amino acid of the position 6 on the NP366 CD8 peptide is crucial for peptide specificity, but not essential for the maintenance of the peptide dominance [11].

Although it was found that PA protein contains peptides that can be recognized by H2-D^b^ molecule and several peptides were predicted as potential candidates [23], [29], the bona fide CD8 epitopes from this protein were not identified until a report that defined the prominence of PA224 CD8 epitope during primary X31 and PR8 infection [3]. In the present study, we found that the predicted PA224 epitope on CA/E3/09 (CAPA224) has one amino acid on position 1 that is different from PA224 of X31 virus (X31PA224). However, this substitution did not affect the ability of the CAPA224 peptide to induce cytokine responses after X31 virus infection as demonstrated by ELISPOT assay. The results support previous conclusion that amino acids of position 5, 6, 7 and 9, but not others on X31PA224 peptide are critical for TCR recognition and activation [3], [12], [28], [30], [31].

In contrast to the robust anti-PA224 CD8 response during X31 infection, we did not observe any response to either CAPA224 or X31PA224 peptide after CA/E3/09 infection. Further, X31PA224 tetramer staining failed to detect any CD8 cells in various tissue samples from CA/E3/09 infected animals. Thus, S224P substitution does not affect the antigenicity of peptide PA224, but abolishes its immunogenicity during CA/E3/09 infection. The exact reason for the loss of CAPA224 dominance is not known at this moment, but it is possible that CAP224 peptides are not processed and recognized by H2-D^b^ after CA/E3/09 infection. The fact that the predicted H2-D^b^ binding for CAPA224 is drastically lower than the X31PA224 supports the possibility that CAP224 is not able to form a stable MHC complex. We have not yet tested this hypothesis. Even though we do not fully understand the molecular basis of the effect of the S to P switch on the PA224 peptide recognition and presentation, further analysis of the first amino acid residue on the MHC complex stability or the established crystal structure of H2-D^b^-PA224 [28], [30] may provide useful hints.

In addition to the CANP366 and CAPA224 epitopes, we examined other predicted epitopes, among which CANP39 elicited a consistent response. Since the frequency of IFN-γ producing cells elicited by peptide CANP39 is slightly over half of that against CANP366, though still at least two-fold higher than the other peptides, we considered CANP39 as subdominant epitope and other peptides including CANP41, CANP55, CANP130, CANP296 and CAPA439 as minor CD8 epitopes for CA/E3/09 virus. These subdominant and minor CD8 epitopes have not been described previously, in spite of the conservation of some of these sequences in X31/PR8 viruses. Thus, our data provided evidence that CA/E3/09 infection results in a diverse usage of the CD8 TCR repertoire and novel hierarchy of CD8 epitopes.

Most importantly, our analysis of X31NP366 and CANP366 specific CD8 T cells using MHC tetramers and ELISPOT showed that a population that is truly cross-reactive to X31NP366 and CANP366 epitopes appeared after CA/E3/09 secondary infection in X31 primed animals, but is not seen in primary infections. Our data suggested that prior priming promotes the generation of cross-reactive NP366 dominant CD8 T cells that can be recognized by both NP366 peptides from X31 and CA/E3/09 viruses. The selective expansion of cross-reactive NP366 cells has also been demonstrated previously using priming and challenge model with X31 or PR8 and NT60, an influenza virus that differs in sequence of the NP366 epitope at position 7 and 8 [32]. Thus, our results confirmed the previous finding that the rare cross-reactive CD8 T cells generated during primary influenza infection could be robustly expanded and protective for the heterologous variant infection. It is not known whether this type of cross-reactivity is unique to the NP366 epitope, but it seems more likely that other examples of this form of reactivity exist. This phenomenon is particularly important in the context of human influenza immunity, as humans may be exposed and primed to many related and unrelated influenza viruses over a lifetime. Thus, it raises questions about whether CD8+ T cell specificities that may be rare in single infections become more prevalent upon repeated infections in human. Previous published study showed cross-reactive NP418 human CD8 T cells could be generated in-vitro and detected with low frequency in stimulated human PBMCs [33], suggesting similar immunological selection operates in human during repeated infection with influenza variants to provide protection. The number of such examples of immune reactivity is not known, but deserves further investigation.

In summary, we have identified and characterized cross-reactive CD8+ T cells that respond against the 2009 H1N1 pandemic influenza virus during primary infection and secondary challenge. The results provided mechanistic explanations for the reported heterologous protection against CA/E3/09 that is observed in human subjects and stimulated in animal models offered by virus priming. Our data is relevant to the design of vaccination strategies against existing and emerging pandemic influenza viruses.

## Materials and Methods

### Ethics Statement

Experiments involving animals were approved by Animal Care and Use Committee at the University of Rochester and complied with the recommendations in the Guide for the Care and Use of Laboratory Animals of the National Institutes of Health.

### Animals

Female C57BL/6 (B6) mice were purchased from The Jackson Laboratory and used for infection between 8 and 12 weeks old. Animals were maintained in the University of Rochester AAALAC certified Vivarium facilities under specific pathogen-free conditions using microisolator technology.

### Viruses and Infection

Influenza A/PR/8/34 (PR8, H1N1) and Influenza A/HKx31 (X31, H3N2) were prepared in eggs as described [34]. Influenza X31 is a recombinant virus containing the HA and NA from a 1968 Hong Kong influenza virus but sharing the internal viral proteins from the PR8 virus. Generation of 2009 pandemic H1N1 virus, CA/E3/09 was reported previously [18]. Virus titer was determined as EID50/ml or PFU/ml. For primary infection, mice were intranasally inoculated with 30 µl of non-lethal doses of viruses (PR8, X31 and CA/E3/09). For rechallenge study, mice were primed with X31 virus and rested for 42 days before 2^nd^ challenge with a lethal dose of CA/E3/09 virus (3000 PFU per mouse).

### CD8 Peptide Prediction and Synthesis

Potential H2-D^b^ restricted 9-mer CD8 peptides from the NP and 10-mer CD8 peptides from the PA proteins of CA/E3/09 virus [18] and X31(PR8) virus (GenBank accession number for NP, ACO94830 and for PA, ACO94833) were generated by NetMHC-3.0 [35] (http://www.cbs.dtu.dk/services/NetMHC-3.0/). 10 peptides from each protein showing highest affinity (Table 1) were synthesized in-house at University of Rochester Medical Center, Rochester, NY. The purity of the synthesized peptides was 96%, as determined by HPLC analysis. Peptides were dissolved in DMSO and stored in the freezer.

### Antibodies and Reagents

Alexa Fluor® 700-conjugated anti-CD3, PerCP-Cy5.5-conjugated anti-CD4 and APC-Cy7-conjugated anti-CD8 were purchased from BioLegend (San Diego, CA). PE-conjugated anti-CD107, FITC-conjugated anti-IFN-γ, and unconjugated anti-CD16/32 were from eBioscience (San Diego, CA). Live/Dead fixable violet fluorescent reactive dye was from Molecular Probes (Invitrogen, Eugene, OR). APC-conjugated tetramer H2-D^b^/influenza X31 NP366–374, PE-conjugated tetramer H2-D^b^/influenza X31 PA224–233 and PE-conjugated H2-D^b^/influenza CA/E3/09 NP366–374 were prepared by the NIH Tetramer Core Facility at Emory University. Phorbol myristate acetate (PMA), Ionomycin, Monensin, and Histopaque 1083 were ordered from Sigma (St. Louis, MO). Fixation and permeabilization were performed using a BD Cytofix/Cytoperm kit from BD Bioscience (San Jose, CA).

### Cell Sample Preparation and Stimulation

Lung, mediastinal lymph node (MLN) and spleen tissues were harvested and minced. All samples were depleted of red blood cells (RBC) by using a buffered ammonium chloride solution for 7 min. Lung single-cell suspensions were obtained by pressing the organs through a 200-gauge wire mesh and filtered through a 90-µm nylon mesh. Lung lymphocytes were isolated at the interface of an 18-min centrifugation step at 1,600×*g* with Histopaque 1083. Cells were counted with trypan blue exclusion. Cell samples were then seeded in a 96-well plate at 0.5×10^6^ cells per well with or without Monensin (5 µg/ml) and stimulated with individual CD8 peptides under final concentration of 5 µg/ml PMAI (phorbol-12-myristate-13-acetate or PMA and Ionomycin) as described before [36]. Incubation was performed at 37°C for 4 h for intracellular cytokine staining or 16 h for ELISPOT assay.

### FACS Staining

Freshly isolated or cultured cells were washed with staining buffer (phosphate-buffered saline plus 1% fetal bovine serum) and blocked with unlabeled anti-CD16/32 for 20 min, followed by staining with Live/Dead violet dye and respective antibodies for 30 min at 4°C. For tetramer staining, cell samples were first stained with different tetramers with optimal concentration for 1 h at room temperature followed by other antibody and Live/Dead staining. For IFN-γ cytokine staining, cells were then fixed with 100 µl of Cytofix/Cytoperm for 10 min at room temperature followed with two washes using permeabilization-wash buffer (perm/wash buffer; BD Biosciences, San Diego, CA). Intracellular staining was performed for 30 min at 4°C. Cells were then washed twice with perm/wash buffer and resuspended in staining buffer before samples were run in the LSRII machine (BD Biosciences, San Jose, CA). All fluorescence-activated cell sorter (FACS) data were analyzed using FlowJo software (Tree Star, San Carlos, CA).

### ELISPOT Assay

Anti mouse IFN-γ antibody (R4-6A2) (BD Pharmingen, San Diego, CA) was diluted into 5 µg/ml for coating in 96-well MultiScreen HTS, IP Elispot plates (Millipore, Billerica, MA). The plates were washed with RPMI medium containing 10% FBS before adding prepared spleen cells (0.5×10^6^ cells/well). The cells were stimulated with each peptide under final concentration of 5 µg/ml for 16 h before washing with PBS-Tween. Biotinated IFN-γ antibody (XMG1.2) (BioLegend, San Diego, CA) was 1∶2000 diluted and added into plate for 1 h incubation at room temperature. The plates were washed and 1∶1000 diluted alkaline phosphatase conjugated streptavidin (Jackson Laboratory, Bar Harbor, ME) added and incubated for 30 min. Finally, the Vector® Blue Alkaline Phosphatase Substrate Kit III (Vector Laboratory, Burlingame, CA) was used to develop spots per manufacturer's instructions. Elispot plates were analyzed using the CTL ImmunoSpot plate reader and counting software (Cellular Technology Limited, Cleveland, OH).

### Statistical Analysis

Two-tailed, unpaired Student *t* test was used to compare the difference between any two appropriate groups. A *p* value of <0.05 was considered statistically significant.
